# Health-related quality of life trajectories up to 15 years after curative treatment for esophageal cancer: a prospective cohort study

**DOI:** 10.1097/JS9.0000000000001026

**Published:** 2023-12-19

**Authors:** Zhao Cheng, Asif Johar, Jesper Lagergren, Anna Schandl, Pernilla Lagergren

**Affiliations:** a Surgical Care Science, Department of Molecular medicine and Surgery, Karolinska Institutet, Karolinska University Hospital, Stockholm, Sweden; b Upper Gastrointestinal Surgery, Department of Molecular medicine and Surgery, Karolinska Institutet, Karolinska University Hospital, Stockholm, Sweden; c School of Cancer and Pharmaceutical Sciences, King’s College London, United Kingdom; d Department of Surgery and Cancer, Imperial College London, United Kingdom

**Keywords:** esophageal neoplasm, patient-reported outcome, risk factor

## Abstract

**Background:**

The differentiation of specific, long-term health-related quality of life (HRQL) trajectories among esophageal cancer survivors remains unclear. The authors aimed to identify potentially distinctly different HRQL-trajectories and uncover the underlying factors of such trajectories in patients having undergone surgery (esophagectomy) for esophageal cancer.

**Materials and methods:**

This nationwide, prospective, and longitudinal cohort study included 420 patients who underwent curative treatment for esophageal cancer, including esophageal cancer surgery, in Sweden from 2001to 2005. The main outcome was HRQL summary score trajectories, measured by the well-validated EORTC QLQ-C30 questionnaire at 6 months, 3, 5, 10, and 15 years after esophagectomy, and analyzed using growth mixture models. Potentially underlying factors for these trajectories (age, sex, education, proxy baseline HRQL, comorbidity, tumor histology, chemo(radio)therapy, pathological tumor stage, and postoperative complications) were analyzed using weighted logistic regression providing odds ratios (OR) with 95% CI.

**Results:**

Four distinct HRQL summary score trajectories were identified: Persistently good, improving, deteriorating, and persistently poor. The odds of belonging to a persistently poor trajectory were decreased by longer education (>12 years versus <9 years: OR 0.18, 95% CI: 0.05–0.66) and adenocarcinoma histology (adenocarcinoma versus squamous cell carcinoma: OR 0.37, 95% CI: 0.16–0.85), and increased by more advanced pathological tumor stage (III–IV versus 0–I: OR 2.82, 95% CI: 1.08–7.41) and postoperative complications (OR 2.94, 95% CI: 1.36–6.36).

**Conclusion:**

Distinct trajectories with persistently poor or deteriorating HRQL were identified after curative treatment for esophageal cancer. Education, tumor histology, pathological tumor stage, and postoperative complications might influence HRQL trajectories. The results may contribute to a more tailored follow-up with timely and targeted interventions. Future research remains to confirm these findings.

## Introduction

Highlights

- Fifteen-year health-related quality of life (HRQL) trajectories after esophagectomy were modeled.
- Vulnerable patients with persistently poor or deteriorating HRQL were identified.
- Factors associated with poor HRQL trajectories were identified.

Esophageal cancer is the seventh most common cancer in terms of incidence and the sixth leading cause of cancer-related mortality globally^1^. It is known for its poor prognosis, with an overall 5-year survival below 20%^2–4^. The mainstay of curative treatment of this biologically aggressive tumor is extensive resection surgery (esophagectomy), often combined with neoadjuvant or perioperative chemo(radio)therapy. Even after the completion of curatively intended treatment, the post-treatment survival is less than 50%^5–7^, and the patient’s health-related quality of life (HRQL) is substantially impaired^8^. This typically includes reduced physical, role, and social functions, as well as several symptoms, for example appetite loss, fatigue, and reflux or regurgitation, which might persist or even deteriorate in the long-term^9–11^.

HRQL is usually measured by validated patient-reported questionnaires to assess subjective perceptions or experiences regarding specific functions and symptoms, as well as general HRQL^12,13^. Previous studies have investigated postoperative HRQL among esophageal cancer patients, which could be influenced by the baseline HRQL level (immediately after diagnosis)^9,14^, and patient-, tumor- and treatment characteristics^15–20^. To our knowledge, no studies have yet identified distinct HRQL trajectories following esophagectomy in esophageal cancer patients and the underlying factors. A longitudinal cohort study is warranted to disentangle the long-term HRQL development and to facilitate the identification of patients at higher risk for adverse outcomes.

In this study, we hypothesize that there are distinct and separable long-term HRQL-trajectories after curative treatment for esophageal cancer and that whether a patient belongs to a specific trajectory depends on certain underlying factors, for example specific patient-, tumor- and treatment characteristics. If these hypotheses are proven true, the findings may help guide healthcare professionals, patients, and family caregivers toward more individualized follow-up and timely interventions.

## Material and methods

### Study design

This population-based and prospective cohort study included 90% of all patients with cancer of the esophagus or gastroesophageal junction (esophageal cancer from here on) who underwent curatively intended esophagectomy, in Sweden between 2nd April 2001 and 31st December 2005. These patients were followed up until 31st December 2020, that is up to 15 years after surgery. All participating patients gave written informed consent. The study was approved by the Regional Ethical Review Board in Stockholm, Sweden. The work has been reported in line with the strengthening the reporting of cohort, cross-sectional and case–control studies in surgery (STROCSS) criteria^21^ (Supplemental Digital Content 1, http://links.lww.com/JS9/B605). This study was registered in the research registry: Clinicaltrials.gov.

### Data collection

The data source used in this study is a Swedish national cohort for esophageal and cardia cancer patients with 97% coverage where patients were identified via a nationwide network of all hospital departments involved in the diagnosis or treatment of esophageal cancer and all six regional cancer centers in Sweden, which has been described elsewhere^22,23^. HRQL was reported by the patients at 6 months, 3, 5, 10, and 15 years after esophagectomy by mailed questionnaires. Up to three reminder letters were sent if necessary. Patients taking part in at least one postoperative HRQL assessment were included in the present study. Vital status was obtained from the National Register of the Total Population. Data on age, sex, tumor histology, treatment, pathological tumor stage, and postoperative complications (within 30 days of surgery) were collected by review of medical records according to a predefined protocol. Education information was retrieved from the Longitudinal Integration Database for Health Insurance and Labor Market (LISA). To obtain proxy baseline HRQL data (before diagnosis), a random sample of the Swedish population (reference population) responded to the same HRQL questionnaire as the patients^24^. Each patient in the study cohort was matched with ~110 individuals from this reference population by age (5-year age groups), sex, education (<9, 9–12, and >12 years of education), and comorbidity (cardiovascular, respiratory, diabetes, and others). The proxy baseline HRQL scores were calculated by the mean scores from the matched individuals. Finally, comorbidity data included in the most well-validated version of the Charlson comorbidity index were extracted from the Swedish National Patient Register^25^. No sampling was done for this exploratory investigation, which is based on a nationwide cohort, hence it was determined that no sample-size calculation was required.

### HRQL outcomes

The main outcome was HRQL-trajectories (categorical variable) based on the HRQL summary score. Secondary outcomes were HRQL-trajectories (categorical variables) based on more specific HRQL aspects. The HRQL questionnaire used was the well-validated European Organization for Research and Treatment of Cancer Quality of Life Questionnaire-Core 30 (EORTC QLQ-C30). This was developed for self-administration to be completed by patients themselves without the involvement of healthcare staff^12,26^. This 30-item cancer-specific questionnaire includes one global quality of life scale, five functional scales (physical, role, emotional, cognitive, and social function), three symptom scales (fatigue, nausea, and pain), and six single symptom items (dyspnea, insomnia, appetite loss, constipation, diarrhea, and financial difficulties). The questionnaire scores were transformed into 0–100 scales. The HRQL summary score was calculated as the mean of 13 of the 15 scales and items in the EORTC QLQ-C30, excluding global quality of life and the financial difficulties scale, and with symptom scores reversed to obtain the same direction as functions^27^. Higher scores in the global quality of life scale, functional scales, and HRQL summary score represent better HRQL or function, while higher scores in the symptom scales or items correspond to more symptoms. Missing HRQL data were handled according to the EORTC scoring manual^27^.

### Underlying factors

The study examined nine predefined factors that might influence HRQL-trajectory category belonging: Age at surgery (continuous variable), sex (female or male), education (<9, 9–12, and >12 years of education), proxy baseline HRQL scores (continuous variable), comorbidity (Charlson comorbidity index score 0, 1, or ≥2, not counting esophageal cancer), tumor histology (squamous cell carcinoma or adenocarcinoma), chemo(radio)therapy (no or yes), pathological tumor stage (0–I, II, or III–IV), and 30-day postoperative complications [no or yes, definitions provided in the Supplementary material (Supplemental Digital Content 2, http://links.lww.com/JS9/B606)]. These factors were selected based on the existing literature^9,14–20^ and availability in the study cohort.

### Statistical analysis

Growth mixture models were used to identify distinct HRQL-trajectories, which allow exploratorily uncovering of homogeneous subgroups within a heterogeneous population^28–31^. Models were fitted with different numbers of trajectories (up to five depending on HRQL scales and items) and model assumptions (different latent or residual variance and linear or quadratic splines). The best models were decided by seven criteria: Akaike Information Criterion (AIC), Bayesian Information Criterion (BIC), sample-size adjusted BIC, entropy, Vuong-Lo-Mendell-Rubin test (VLMR), adjusted Lo-Mendell-Rubin test (aLMR), and the trajectory interpretability^32^. AIC, BIC, and sample-size adjusted BIC compare the log-likelihood of nested models, where smaller values indicate a better fit. Entropy represents the uncertainty of trajectory categorization, where higher values represent higher accuracy. VLMR and aLMR compare the K-trajectory model with the (K-1)-trajectory model and a *P*-value below 0.05 signifies the fit of the K-trajectory model. Only models in which each trajectory included at least 15% (based on estimated posterior probabilities) of the patients were considered. The trajectory category probabilities for each patient were derived from these models. Patients were assigned to the trajectory category with the highest estimated posterior probability. The model-estimated and sample-observed mean for each trajectory were presented. The sample-observed means were calculated as weighted means of the HRQL scores in each trajectory with their probability of trajectory category as weights.

Weighted binomial and multinomial logistic regression models were fitted to calculate odds ratios (OR) with 95% CI for associations between potential underlying factors and HRQL-trajectory category with complete case analyses^30^. All nine selected factors were included in the multivariable models and assumed to be potential confounders for each other. The trajectory category probabilities from growth mixture models were used as weights in the logistic regression analyses in order to account for potential misclassification. The reference groups were the trajectories representing the best HRQL scores. Statistical significance level was set at 5% level with a two-sided test. The MPlus version 8.7 software (Los Angeles, California: Muthén & Muthén) was used for growth mixture model analyses, and SAS version 9.4 software (Cary, North Carolina: SAS Institute Inc.) was used for all other analyses.

## Results

### Patients

The cohort included 616 esophageal cancer patients who underwent esophagectomy (predominantly open thoraco-abdominal Ivor-Lewis procedure), between 2 April 2001 and 31 December 2005, in Sweden. At 6 months, 3, 5, 10, and 15 years after surgery, 506 (82%), 212 (34%), 153 (25%), 104 (17%), and 70 (11%) patients were alive, of whom 402 (79%), 178 (84%), 141 (92%), 92 (88%), and 52 (74%) completed the HRQL questionnaires, respectively. Among these, the 420 patients who responded to at least one of the postoperative HRQL measurements constituted the final study cohort (Supplementary material Table 1, Supplemental Digital Content 2, http://links.lww.com/JS9/B606). The mean age was 65.5 years, and the majority were men (81.2%), had a Charlson comorbidity score of 0 (57.6%), a tumor of adenocarcinoma histology (76.2%), and did not suffer any 30-day postoperative complication (65.5%).

### Presence of HRQL-trajectories

**Figure 1:**
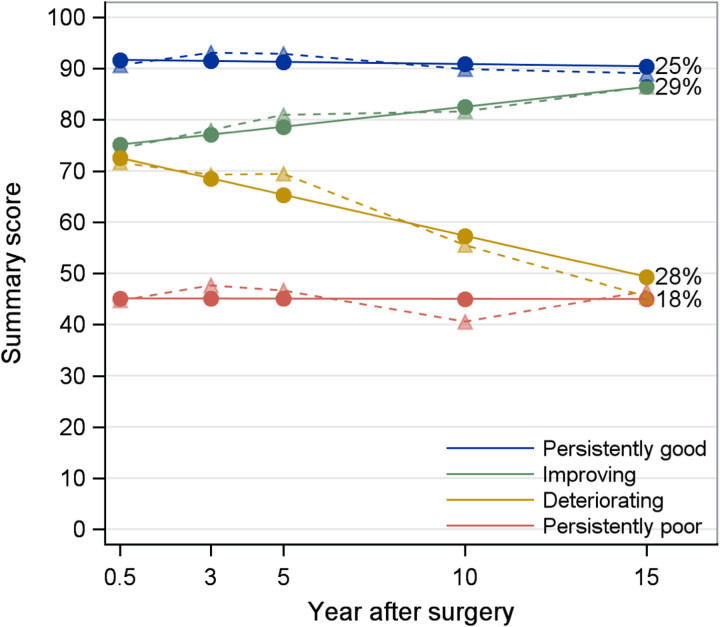
Summary score trajectories of health-related quality of life after surgery for esophageal cancer. Solid lines represent model-estimated mean, and dash lines represent sample-observed mean. The percentage after each trajectory is the final patient proportion for the trajectory category based on the most likely latent class membership.

For the main outcome, HRQL summary scores, four distinct trajectories were identified, which were labeled ‘persistently good’, ‘improving’, ‘deteriorating’, and ‘persistently poor’ (Fig. 1). Fit statistics for model comparison are presented in Supplementary Material Tables 2–4 (Supplemental Digital Content 2, http://links.lww.com/JS9/B606). Patients’ characteristics within each of the four trajectories are presented in Table 1. Patients with low education levels, tumors of squamous cell carcinoma histology, advanced pathological tumor stage, and postoperative complications were overrepresented in the persistently poor trajectory compared to the other three trajectories.

**Table 1:** Characteristics of 420 patients who had at least one measurement of health-related quality of life (HRQL) by summary score trajectory.

	HRQL summary score trajectory			
	Persistently good number (%)	Improving number (%)	Deteriorating number (%)	Persistently poor number (%)
Age				
Mean (SD)	66.6 (9.1)	64.4 (9.8)	66.7 (8.7)	64.5 (10.8)
Sex				
Female	22 (17.7)	22 (16.8)	17 (19.5)	18 (23.1)
Male	102 (82.3)	109 (83.2)	70 (80.5)	60 (76.9)
Education (years)				
<9	47 (37.9)	57 (43.5)	35 (40.2)	46 (59.0)
9–12	54 (43.5)	47 (35.9)	40 (46.0)	24 (30.8)
>12	21 (16.9)	24 (18.3)	11 (12.6)	5 (6.4)
Missing	2 (1.6)	3 (2.3)	1 (1.1)	3 (3.8)
Proxy baseline HRQL summary score				
Mean (SD)	88.9 (5.4)	88.2 (6.1)	88.4 (6.5)	86.4 (7.4)
Charlson comorbidity index				
0	76 (61.3)	73 (55.7)	46 (52.9)	47 (60.3)
1	29 (23.4)	39 (29.8)	19 (21.8)	18 (23.1)
≥2	19 (15.3)	19 (14.5)	22 (25.3)	13 (16.7)
Tumor histology				
Squamous cell carcinoma	19 (15.3)	33 (25.2)	20 (23.0)	28 (35.9)
Adenocarcinoma	105 (84.7)	98 (74.8)	67 (77.0)	50 (64.1)
Chemo (radio)therapy				
No	120 (96.8)	122 (93.1)	82 (94.3)	71 (91.0)
Yes	4 (3.2)	9 (6.9)	5 (5.7)	7 (9.0)
Pathological tumor stage				
0–I	32 (25.8)	36 (27.5)	19 (21.8)	9 (11.5)
II	32 (25.8)	40 (30.5)	31 (35.6)	21 (26.9)
III–IV	58 (46.8)	54 (41.2)	36 (41.4)	47 (60.3)
Missing	2 (1.6)	1 (0.8)	1 (1.1)	1 (1.3)
Postoperative complications				
No	97 (78.2)	83 (63.4)	49 (56.3)	46 (59.0)
Yes	27 (21.8)	48 (36.6)	38 (43.7)	32 (41.0)

**Figure 2:**
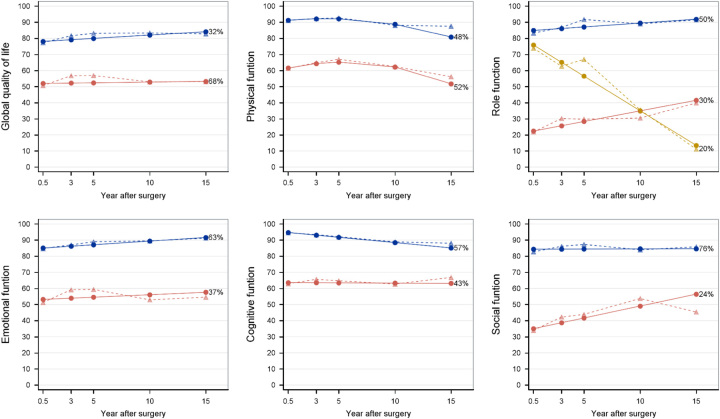
Global quality of life and functional scales trajectories after surgery for esophageal cancer. Solid lines represent model-estimated mean, and dash lines represent sample-observed mean. The percentage after each trajectory is the final patient proportion for the trajectory category based on estimated posterior probabilities.

**Figure 3:**
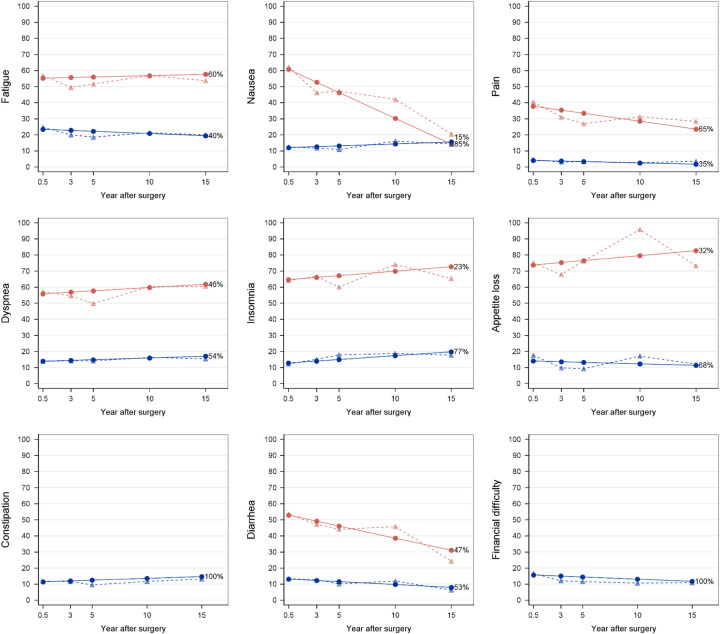
Symptom scales and items trajectories after surgery for esophageal cancer. Solid lines represent model-estimated mean, and dash lines represent sample-observed mean. The percentage after each trajectory is the final patient proportion for the trajectory category based on estimated posterior probabilities.

Among the secondary outcomes, the global quality of life scale and most functional scales had two distinct trajectories, except for the role function, which had three trajectories (Fig. 2). Apart from starting from different scores at the 6-month measurement, global quality of life and most functions were stable within each trajectory category over time, especially for the persistently good trajectories (blue trajectories in Fig. 2). The mean scores of physical function trajectories decreased by about 10 during the last 5 years of follow-up in both the persistently good and poor trajectories. A deteriorating trajectory regarding role function was found among 20% of the patients, with the mean score decreasing from about 75 to 15 during the study period. Improving trajectories for the role and social function were seen among 30 and 24% of patients, respectively (Fig. 2). There were two distinct trajectories identified for most symptom scales and items, except for constipation and financial difficulties where only one trajectory was identified (Fig. 3). Persistently good trajectories with fewer symptoms (blue trajectories in Fig. 3) were identified for all measured symptoms. Improving trajectories for nausea, pain, and diarrhea were seen among 15, 65, and 47% of patients, respectively (Fig. 3).

### Factors associated with HRQL-trajectories

Patients with longer education had lower odds of having a persistently poor HRQL summary score trajectory (9–12 versus <9 years: OR 0.46, 95% CI: 0.21–0.98; >12 versus <9 years: OR 0.18, 95% CI: 0.05–0.66) (Table 2). Compared to patients with a history of squamous cell carcinoma, those with adenocarcinoma showed lower odds of belonging to the persistently poor trajectory (OR 0.37, 95% CI: 0.16–0.85). Pathological tumor stages III–IV was associated with higher odds of a persistently poor trajectory, compared to stages 0–I (OR 2.82, 95% CI: 1.08–7.41). Patients with 30-day postoperative complications were almost three times more likely to have a deteriorating trajectory (OR 2.91, 95% CI: 1.36–6.21) and persistently poor trajectory (OR 2.94, 95% CI: 1.36–6.36), compared to those without such complications. No statistically significant associations were found for age, sex, proxy baseline HRQL summary score, comorbidity, or chemo(radio)therapy in relation to the HRQL summary score trajectory belonging (Table 2).

**Table 2:** Odds ratios (95% CI) between factors and health-related quality of life (HRQL) summary score trajectory after surgery for esophageal cancer.

	HRQL summary score trajectory		
	Persistently good (reference)^a^		
	Improving	Deteriorating	Persistently poor
Age			
Continuous	0.97 (0.94–1.01)	1.01 (0.97–1.05)	0.98 (0.94–1.02)
Sex			
Female	1.00 (Reference)	1.00 (Reference)	1.00 (Reference)
Male	1.15 (0.50–2.66)	0.95 (0.38–2.35)	0.82 (0.33–2.06)
Education (years)			
<9	1.00 (Reference)	1.00 (Reference)	1.00 (Reference)
9–12	0.64 (0.32–1.28)	1.09 (0.51–2.35)	**0.46** (**0.21**–**0.98)**
>12	0.80 (0.33–1.91)	0.65 (0.22–1.98)	**0.18** (**0.05**–**0.66)**
Proxy baseline HRQL summary score			
Continuous	1.00 (0.95–1.06)	0.98 (0.93–1.05)	0.95 (0.89–1.01)
Charlson comorbidity index			
0	1.00 (Reference)	1.00 (Reference)	1.00 (Reference)
1	1.33 (0.63–2.80)	1.11 (0.46–2.66)	0.75 (0.31–1.86)
≥2	1.00 (0.40–2.48)	1.55 (0.61–3.94)	0.88 (0.32–2.40)
Tumor histology			
Squamous cell carcinoma	1.00 (Reference)	1.00 (Reference)	1.00 (Reference)
Adenocarcinoma	0.67 (0.31–1.48)	0.74 (0.30–1.80)	**0.37** (**0.16**–**0.85)**
Chemo(radio)therapy			
No	1.00 (Reference)	1.00 (Reference)	1.00 (Reference)
Yes	1.83 (0.41–8.10)	1.25 (0.21–7.60)	2.71 (0.55–13.29)
Pathological tumor stage			
0–I	1.00 (Reference)	1.00 (Reference)	1.00 (Reference)
II	1.14 (0.51–2.54)	1.72 (0.69–4.27)	1.92 (0.66–5.52)
III–IV	0.79 (0.37–1.67)	1.02 (0.43–2.45)	**2.82** (**1.08**–**7.41)**
Postoperative complications			
No	1.00 (Reference)	1.00 (Reference)	1.00 (Reference)
Yes	1.89 (0.94–3.79)	**2.91** (**1.36–6.21)**	**2.94** (**1.36–6.36)**

a The reference trajectory is the persistently good trajectory in Figure 1.

Bold results are statistically significant.

For secondary outcomes, longer education was associated with reduced odds of having poor trajectories regarding global quality of life (>12 versus <9 years: OR 0.42, 95% CI: 0.21–0.85), physical function (>12 versus <9 years: OR 0.43, 95% CI: 0.22–0.85), role function (>12 versus <9 years: OR 0.37, 95% CI: 0.17–0.82), emotional function (9–12 versus <9 years: OR 0.59, 95% CI: 0.35–0.98) (Table 3), as well as lower odds of belonging to a trajectory with more problems with pain (9–12 versus <9 years: OR 0.61, 95% CI: 0.37–0.99) and insomnia (>12 versus <9 years: OR 0.38, 95% CI: 0.16–0.90) (Table 4). Compared to patients with squamous cell carcinoma, those with adenocarcinoma had lower odds of poor trajectories for physical function (OR 0.45, 95% CI: 0.27–0.77), role function (OR 0.52, 95% CI: 0.29–0.95), and dyspnea (OR 0.49, 95% CI: 0.29–0.82). A more advanced tumor stage was associated with higher odds of belonging to poor trajectories regarding global quality of life (II versus 0–I: OR 2.21, 95% CI: 1.14–4.27), role function (II versus 0–I: OR 3.83, 95% CI: 1.72–8.50; III–IV versus 0–I: OR 5.64, 95% CI: 2.67–11.91), social function (III–IV versus 0–I: OR 2.59, 95% CI: 1.29–5.21), and appetite loss (III–IV versus 0–I: OR 3.19, 95% CI: 1.68–6.05). Patients with postoperative complications had increased odds of having poor trajectories for poor global quality of life (OR 2.42, 95% CI: 1.39–4.22), physical function (OR 1.77, 95% CI: 1.12–2.82), role function (OR 2.50, 95% CI: 1.46–4.27), emotional function (OR 1.84, 95% CI: 1.13–3.00), fatigue (OR 2.24, 95% CI: 1.37–3.66), nausea (OR 3.42, 95% CI: 1.86–6.29), dyspnea (OR 2.07, 95% CI: 1.29–3.31), insomnia (OR 1.79, 95% CI: 1.07–2.98), and appetite loss (OR 2.49, 95% CI: 1.53–4.04). No statistically significant associations were found between the factors sex, proxy baseline HRQL score, comorbidity, or chemo(radio)therapy in relation to global quality of life or functional trajectory belonging (Table 3), and no associations were found between age, sex, or chemo(radio)therapy in relation to symptom trajectory belonging (Table 4).

**Table 3:** Odds ratios (95% CIs) between factors and health-related quality of life (HRQL) trajectories of global quality of life and functional scales after surgery for esophageal cancer.

	Global quality of life	Physical function	Role function		Emotional function	Cognitive function	Social function
	Persistently good (reference)^a^	Persistently good (reference)^a^	Persistently good (reference)^a^		Persistently good (reference)^a^	Persistently good (reference)^a^	Persistently good (reference)^a^
	Persistently poor	Persistently poor	Deteriorating	Poor and improving	Persistently poor	Persistently poor	Poor and improving
Age							
Continuous	1.00 (0.98–1.03)	1.03 (1.00–1.05)	1.01 (0.97–1.06)	**0.97** (**0.95–1.00)**	0.98 (0.95–1.01)	0.99 (0.97–1.02)	**0.96** (**0.94–0.99)**
Sex							
Female	1.00 (Reference)	1.00 (Reference)	1.00 (Reference)	1.00 (Reference)	1.00 (Reference)	1.00 (Reference)	1.00 (Reference)
Male	0.86 (0.45–1.66)	0.85 (0.47–1.52)	1.08 (0.40–2.92)	1.28 (0.64–2.57)	0.98 (0.52–1.83)	0.97 (0.56–1.69)	1.40 (0.69–2.86)
Education (years)							
<9	1.00 (Reference)	1.00 (Reference)	1.00 (Reference)	1.00 (Reference)	1.00 (Reference)	1.00 (Reference)	1.00 (Reference)
9–12	0.83 (0.48–1.45)	0.71 (0.44–1.15)	1.46 (0.63–3.41)	0.57 (0.33–1.01)	**0.59** (**0.35–0.98)**	0.70 (0.43–1.13)	0.81 (0.47–1.41)
>12	**0.42** (**0.21–0.85)**	**0.43** (**0.22–0.85)**	0.57 (0.15–2.14)	**0.37** (**0.17–0.82)**	0.52 (0.25–1.08)	0.65 (0.34–1.25)	0.63 (0.29–1.39)
Proxy baseline HRQL score^b^							
Continuous	0.98 (0.95–1.00)	0.98 (0.95–1.01)	0.97 (0.93–1.01)	0.99 (0.97–1.02)	0.99 (0.94–1.03)	0.97 (0.93–1.00)	1.01 (0.97–1.05)
Charlson comorbidity index							
0	1.00 (Reference)	1.00 (Reference)	1.00 (Reference)	1.00 (Reference)	1.00 (Reference)	1.00 (Reference)	1.00 (Reference)
1	0.97 (0.53–1.78)	1.11 (0.64–1.91)	1.75 (0.70–4.39)	1.29 (0.68–2.42)	0.67 (0.37–1.21)	0.82 (0.48–1.39)	1.30 (0.70–2.40)
≥2	1.33 (0.65–2.75)	1.60 (0.87–2.95)	1.52 (0.53–4.35)	1.45 (0.73–2.89)	0.75 (0.39–1.46)	1.33 (0.75–2.38)	1.38 (0.68–2.77)
Tumor histology							
Squamous cell carcinoma	1.00 (Reference)	1.00 (Reference)	1.00 (Reference)	1.00 (Reference)	1.00 (Reference)	1.00 (Reference)	1.00 (Reference)
Adenocarcinoma	0.66 (0.36–1.23)	**0.45** (**0.27–0.77)**	0.59 (0.23–1.52)	**0.52** (**0.29–0.95)**	0.61 (0.35–1.05)	0.73 (0.44–1.22)	0.71 (0.40–1.26)
Chemo (radio)therapy							
No	1.00 (Reference)	1.00 (Reference)	1.00 (Reference)	1.00 (Reference)	1.00 (Reference)	1.00 (Reference)	1.00 (Reference)
Yes	2.89 (0.77–10.93)	1.00 (0.41–2.47)	0.47 (0.06–4.00)	1.13 (0.42–3.08)	1.63 (0.64–4.16)	0.77 (0.31–1.94)	2.30 (0.91–5.84)
Pathological tumor stage							
0–I	1.00 (Reference)	1.00 (Reference)	1.00 (Reference)	1.00 (Reference)	1.00 (Reference)	1.00 (Reference)	1.00 (Reference)
II	**2.21** (**1.14–4.27)**	1.49 (0.82–2.69)	0.97 (0.37–2.53)	**3.83** (**1.72–8.50)**	1.81 (0.94–3.49)	0.87 (0.48–1.56)	1.57 (0.73–3.38)
III–IV	1.77 (0.98–3.22)	1.18 (0.68–2.06)	0.67 (0.25–1.76)	**5.64** (**2.67–11.91)**	1.51 (0.82–2.80)	1.10 (0.64–1.89)	**2.59** (**1.29–5.21)**
Postoperative complications							
No	1.00 (Reference)	1.00 (Reference)	1.00 (Reference)	1.00 (Reference)	1.00 (Reference)	1.00 (Reference)	1.00 (Reference)
Yes	**2.42** (**1.39–4.22)**	**1.77** (**1.12–2.82)**	1.45 (0.64–3.31)	**2.50** (**1.46–4.27)**	**1.84** (**1.13–3.00)**	1.44 (0.92–2.25)	1.35 (0.80–2.27)

a The reference trajectory of each scale is the corresponding blue trajectory with persistently good functions in Figure 2.

b The proxy baseline HRQL scores are the corresponding baseline scores of global quality of life or each functional scale.

Bold results are statistically significant.

**Table 4:** Odds ratios (95% CIs) between factors and health-related quality of life (HRQL) trajectories of symptom scales and items^a^ after surgery for esophageal cancer.

	Fatigue	Nausea	Pain	Dyspnea	Insomnia	Appetite loss	Diarrhea
	Persistently less (reference)^b^	Persistently less (reference)^b^	Persistently less (reference)^b^	Persistently less (reference)^b^	Persistently less (reference)^b^	Persistently less (reference)^b^	Persistently less (reference)^b^
	Persistently more	More and alleviating	More and alleviating	Persistently more	Persistently more	Persistently more	More and alleviating
Age							
Continuous	1.01 (0.99–1.04)	0.97 (0.94–1.00)	0.98 (0.96–1.01)	1.00 (0.97–1.02)	0.98 (0.96–1.01)	1.02 (0.99–1.05)	0.99 (0.97–1.02)
Sex							
Female	1.00 (Reference)	1.00 (Reference)	1.00 (Reference)	1.00 (Reference)	1.00 (Reference)	1.00 (Reference)	1.00 (Reference)
Male	0.89 (0.49–1.63)	0.52 (0.26–1.07)	1.18 (0.66–2.11)	1.15 (0.63–2.09)	0.66 (0.35–1.27)	0.92 (0.50–1.69)	1.07 (0.59–1.94)
Formal education (years)							
<9	1.00 (Reference)	1.00 (Reference)	1.00 (Reference)	1.00 (Reference)	1.00 (Reference)	1.00 (Reference)	1.00 (Reference)
9–12	0.70 (0.43–1.15)	0.71 (0.37–1.36)	**0.61** (**0.37–0.99)**	0.88 (0.53–1.45)	0.70 (0.40–1.21)	0.94 (0.57–1.56)	0.86 (0.52–1.41)
>12	0.60 (0.31–1.18)	0.36 (0.13–1.00)	0.55 (0.28–1.06)	0.54 (0.27–1.11)	**0.38** (**0.16–0.90)**	0.66 (0.31–1.39)	0.75 (0.38–1.49)
Proxy baseline HRQL score^c^							
Continuous	1.02 (0.99–1.05)	1.02 (0.92–1.13)	1.01 (0.99–1.03)	1.01 (0.99–1.04)	1.00 (0.98–1.03)	1.05 (0.99–1.12)	**1.06** (**1.01–1.12)**
Charlson comorbidity index							
0	1.00 (Reference)	1.00 (Reference)	1.00 (Reference)	1.00 (Reference)	1.00 (Reference)	1.00 (Reference)	1.00 (Reference)
1	1.60 (0.90–2.84)	0.87 (0.42–1.82)	0.85 (0.50–1.46)	1.18 (0.67–2.10)	0.74 (0.39–1.42)	0.69 (0.39–1.22)	1.25 (0.73–2.14)
≥2	1.26 (0.67–2.40)	0.79 (0.34–1.82)	0.97 (0.53–1.79)	**2.08** (**1.11–3.89)**	1.21 (0.63–2.36)	0.79 (0.42–1.48)	0.83 (0.44–1.58)
Tumor histology							
Squamous cell carcinoma	1.00 (Reference)	1.00 (Reference)	1.00 (Reference)	1.00 (Reference)	1.00 (Reference)	1.00 (Reference)	1.00 (Reference)
Adenocarcinoma	0.61 (0.35–1.05)	1.16 (0.58–2.33)	0.65 (0.38–1.13)	**0.49** (**0.29–0.82)**	0.76 (0.42–1.35)	0.63 (0.37–1.08)	0.91 (0.53–1.54)
Chemo (radio) therapy							
No	1.00 (Reference)	1.00 (Reference)	1.00 (Reference)	1.00 (Reference)	1.00 (Reference)	1.00 (Reference)	1.00 (Reference)
Yes	1.19 (0.45–3.14)	1.89 (0.67–5.33)	1.16 (0.43–3.13)	1.33 (0.52–3.40)	0.76 (0.26–2.23)	1.83 (0.73–4.62)	1.36 (0.55–3.40)
Pathological tumor stage							
0–I	1.00 (Reference)	1.00 (Reference)	1.00 (Reference)	1.00 (Reference)	1.00 (Reference)	1.00 (Reference)	1.00 (Reference)
II	1.67 (0.90–3.10)	0.95 (0.40–2.25)	1.80 (0.99–3.27)	1.09 (0.58–2.03)	0.94 (0.47–1.87)	2.00 (1.00–4.03)	0.68 (0.38–1.25)
III–IV	1.55 (0.88–2.74)	1.50 (0.70–3.18)	1.60 (0.92–2.76)	1.11 (0.62–1.96)	1.06 (0.56–1.99)	**3.19** (**1.68–6.05)**	**0.50** (**0.29–0.88)**
Postoperative complications							
No	1.00 (Reference)	1.00 (Reference)	1.00 (Reference)	1.00 (Reference)	1.00 (Reference)	1.00 (Reference)	1.00 (Reference)
Yes	**2.24** (**1.37–3.66)**	**3.42** (**1.86–6.29)**	1.22 (0.76–1.96)	**2.07** (**1.29–3.31)**	**1.79** (**1.07–2.98)**	**2.49** (**1.53–4.04)**	1.23 (0.77–1.98)

a ‘Constipation’ and ‘Financial difficulty’ are not included in the analysis since only one trajectory was identified.

b The reference trajectory of each scale is the corresponding blue trajectory with persistently fewer symptoms in Figure 3.

c The proxy baseline HRQL scores are the corresponding baseline scores of each symptom scale or item.

Bold results are statistically significant.

## Discussion

This study identified four distinct long-term trajectories of HRQL summary scores among esophageal cancer survivors, as well as separable HRQL-trajectories for global quality of life, certain functions, and specific symptoms. Longer education, adenocarcinoma histology, earlier tumor stage, and no 30-day postoperative complications decreased the odds of having poor HRQL-trajectories.

To the best of our knowledge, this is the first study examining distinct HRQL-trajectories in patients with esophageal cancer. Among the four HRQL summary score trajectories identified, two were stable between 6 months and 15 years after surgery, one persistently good, and one persistently poor. Such persistent trajectories were also found for global quality of life, all functional scales, and all symptoms in more than half of the patients. These findings suggest that HRQL measurements 6 months after treatment often mirror very long-term HRQL-trajectories. Another study following up esophageal cancer patients after esophagectomy also found that the average HRQL did not improve from levels 1 year after surgery compared to patients up to 23 years after esophagectomy^33^. One study followed breast cancer patients for 10 years after diagnosis and found that more than 70% of the patients showed stable (score fluctuation <10) development of both mental and physical HRQL component scores^34^. Whether the HRQL level is truly persistent is unknown. It could also be a result of the combination of aging, response shift (recalibration, reprioritization, and reconceptualization)^35^, increasing disease burden affecting the nature of changing HRQL trajectories. Intensified follow-up with targeted interventions in patients reporting initially poor HRQL levels may prevent them from remaining in a persistently poor trajectory.

The better HRQL-trajectories among patients with longer education and adenocarcinoma histology could be due to lifestyle factors, including lower rates of alcohol abuse, tobacco smoking, and comorbidity. The proxy baseline HRQL scores did not influence the HRQL-trajectory of esophageal cancer survivors in this study. This contrasts with patients with Hodgkin lymphoma^14,31^, but those patients have better survival and less morbidity compared to esophageal cancer patients. The survivorship of patients with a history of esophageal cancer might be more heavily influenced by tumor-related and treatment-related factors. Esophageal cancer patients with earlier pathological tumor stages have a lower fear of cancer recurrence, which may explain their better HRQL-trajectory in the present study. Postoperative complications are associated with more symptoms, for example dyspnea, insomnia and appetite loss, fatigue, and more psychological burden during the recovery^11,36,37^. Thus, the negative influence of postoperative complications on long-term HRQL trajectories seen in this study was not unexpected.

Our findings enable the identification of specific subgroups of esophageal cancer patients who are at a higher risk of experiencing poor HRQL. This includes individuals with both poor initial HRQL and those with factors indicating an increased likelihood of persistent poor HRQL-trajectory. For patients with poor initial HRQL, early identification of challenges and prompt interventions can be integrated into routine follow-up protocols. For patients with factors contributing to persistently poor HRQL, healthcare providers can use this information to target interventions more precisely and proactively, tailoring their approach to the needs of these high-risk subgroups. An intervention with individualized care plans that consider the needs and preferences of each individual patient, ensuring a personalized and patient-centered approach to follow-up and care using modern technology may be a way forward.

The study’s strengths include the nationwide and population-based design with high participating rates, long (up to 15 years) and complete follow-up, use of a well-validated HRQL questionnaire, and data on a large number of factors obtained from medical records and well-validated registers. The weaknesses of this study should also be acknowledged. There have been changes in clinical practice during the long study period. Specifically, the increased use of neoadjuvant chemo(radio)therapy and minimally invasive surgery, which could influence the study’s generalizability. However, no differences have been found regarding long-term HRQL comparing patients with or without neoadjuvant chemo(radio)therapy^38,39^ or minimally invasive versus open surgery^40^. A risk of unmeasured or residual confounding is inherent in observational studies. Such error was counteracted by adjustment for several factors, but data on other potentially relevant factors were not available, for example lifestyle habits, psychosocial variables, family history, or genetic factors. Besides, the HRQL-trajectory might also be influenced by cancer recurrence and palliative treatments, but such data were unavailable in this study. It was not feasible to obtain study patients’ baseline HRQL measures, that is before the esophageal cancer diagnosis, and any HRQL measurement conducted between diagnosis and surgery would be heavily influenced by the awareness and impact of the tumor. We dealt with this issue by using proxy baseline HRQL scores from a matched reference population. Finally, possible misclassification by assigning patients into trajectories according to the posterior probabilities from growth mixture models might also bias the results^41^. To account for this, weighted logistic regression was used, where patients with higher probabilities of the outcome contributed more to the estimation and vice versa. Given the limitations of the study, future studies remain to confirm the findings, particularly those examining other HRQL measurements, with larger sample sizes, and other potentially relevant factors.

## Conclusion

This comprehensive population-based cohort study revealed distinctly different 15-year HRQL-trajectories among esophageal cancer survivors after esophagectomy, highlighting the importance of understanding how specific HRQL functions or symptoms change over time after treatment. Education, tumor histology, pathological tumor stage, and postoperative complications seem to influence HRQL-trajectory belonging. This new knowledge could help guide healthcare providers to a more intensified and tailored follow-up with targeted interventions for esophageal cancer patients with poor initial HRQL and those with factors contributing to persistently poor HRQL-trajectories.

## Ethical approval

The study was approved by the Regional Ethical Review Board in Stockholm, Sweden (diary number 2015/0091–32).

## Consent

The study was performed in accordance with the Declaration of Helsinki. Informed consent was obtained from each participant.

Written informed consent was obtained from the patient for publication of this case report and accompanying images. A copy of the written consent is available for review by the Editor-in-Chief of this journal on request.

## Sources of funding

This study is funded by the Swedish Cancer Society, the Swedish Research Council, and the Cancer Research Funds of Radiumhemmet. Pernilla Lagergren is supported by NIHR Imperial Biomedical Research Centre (BRC) for her Imperial College London affiliation. The funding sources had no role in the study.

## Author contribution

Z.C. and P.L.: study concept; J.L. and P.L.: data acquisition; A.J.: data management; Z.C. and A.J.: statistical analysis; Z.C.: manuscript writing. All authors contributed in study design and manuscript revision.

## Conflicts of interest disclosure

The authors declares no conflicts of interest.

## Research registration unique identifying number (UIN)

ClinicalTrials.gov Identifier: NCT06031155.

## Guarantor

Professor Pernilla Lagergren (ORCID: 0000-0002-4634-0233), Department of Molecular medicine and Surgery, Karolinska Institutet, Retzius Street 13A, 4th Floor, Stockholm, 171 77, Sweden (e-mail: pernilla.lagergren@ki.se).

## Data availability statement

The data of this study are not publicly available due to ethical restrictions. The data are available from the corresponding author P.L. on reasonable request.

## Provenance and peer review

Not commissioned, externally peer-reviewed.

## Supplementary Material

SUPPLEMENTARY MATERIAL
