# Supplementary material for: Health-related quality of life trajectories up to 15 years after curative treatment for esophageal cancer: a prospective cohort study
Source: Int J Surg. 2023 Dec 19;110(3):1537–45. doi: 10.1097/JS9.0000000000001026 (PMC10942160; doi:10.1097/JS9.0000000000001026)
Supplement: SUPPLEMENTARY MATERIAL [file js9-110-1537-s002.docx]

**Title:** **Health-related quality of life trajectories up to 15 years after** **curative treatment for esophageal cancer - a prospective cohort study**

**Supplementary Materials - Index**

| **Supplementary Methods** |  |
| --- | --- |
| The definition of postoperative complications | *pag. 2* |
| **Supplementary Results** |  |
| eTable 1. Characteristics of 420 patients who had at least one measurement of health-related quality of life (HRQL) after surgery for esophageal cancer | *pag. 3* |
| eTable 2. Fit statistics for model comparison of EORTC-QLQ C30 summary score | *pag. 4* |
| eTable 3. Fit statistics for model comparison of EORTC-QLQ C30 global quality of life and functional scales | *pag. 5* |
| eTable 4. Fit statistics for model comparison of EORTC-QLQ C30 symptom scales and items | *pag. 7* |

**Supplementary Methods**

**The definition of postoperative complications:**

Postoperative complications (no or yes) were defined as deviations from the normal postoperative course within 30 days of surgery. The included complications (with definitions) were: postoperative bleeding (>2000 mL or requiring reoperation), anastomotic insufficiency (clinically significant or radiologically detected), substitute necrosis (clinically significant ischemia with ulceration or perforation), intra-abdominal abscess (≥3*3cm radiologically or surgically detected abscess with clinical symptoms such as fever or pain), intrathoracic abscess or empyema (≥3*3cm radiologically or surgically detected abscess with clinical symptoms such as fever, pain or dyspnea); sepsis (causing clinical symptoms such as fever, chills and proven bacteria in the blood), wound infection (causing clinical symptoms and requiring treatment), wound dehiscence (clinically obvious wound rupture), renal failure (dialysis needed), respiratory insufficiency (reintubation or mechanical ventilation needed), hepatic insufficiency (progressive jaundice), recurrent laryngeal nerve paralysis (laryngeal inspection ascertained), pneumonia (radiologically detected infiltrate with clinical symptoms such as fever, cough or dyspnea), pulmonary embolism (radiologically detected), other embolism (radiologically detected and requiring treatment, deep venous thrombosis (radiologically or clinically verified with treatment needs), ileus (radiologically detected ileus in need of surgery), thoracic ductus injury (thoracic lymph leakage requiring drainage for more than 7 days or reoperation), myocardial infarction (electrocardiogram or cardiac enzymes verified), atrial fibrillation (newly electrocardiogram detected and treatment required), cerebral infarction (radiologically verified), strictures in anastomosis (endoscopic intervention required), gastric perforation (surgical intervention required), and other complications.

**Supplementary Results**

| **eTable 1.**  **Characteristics of 420 patients who had at least one measurement of health-related quality of life (HRQL) after surgery for esophageal cancer** | | |
| --- | --- | --- |
|  | Number | % |
| **Age** |  |  |
| Mean (standard deviation) | 65.5 (9.6) | - |
| **Sex** |  |  |
| Female | 79 | 18.8 |
| Male | 341 | 81.2 |
| **Formal education (years)** |  |  |
| <9 | 185 | 44.0 |
| 9-12 | 165 | 39.3 |
| >12 | 61 | 14.5 |
| Missing | 9 | 2.1 |
| **Proxy baseline HRQL summary score** | |  |
| Mean (standard deviation) | 88.1 (6.3) | - |
| **Charlson comorbidity index** |  |  |
| 0 | 242 | 57.6 |
| 1 | 105 | 25.0 |
| ≥2 | 73 | 17.4 |
| **Tumor histology** |  |  |
| Squamous cell carcinoma | 100 | 23.8 |
| Adenocarcinoma | 320 | 76.2 |
| **Chemo(radio)therapy** |  |  |
| No | 395 | 94.0 |
| Yes | 25 | 6.0 |
| **Pathological tumor stage** |  |  |
| 0-I | 96 | 22.9 |
| II | 124 | 29.5 |
| III-IV | 195 | 46.4 |
| Missing | 5 | 1.2 |
| **Postoperative complications** |  |  |
| No | 275 | 65.5 |
| Yes | 145 | 34.5 |
|  | | |

| **eTable 2. Fit statistics for model comparison of EORTC-QLQ C30^a^ summary score** | | | | | | | | | | | |
| --- | --- | --- | --- | --- | --- | --- | --- | --- | --- | --- | --- |
| **Health-related quality of life** | **Trajectory number** | **Latent variance** | **Latent mean** | **Residual variance** | **AIC^b^** | **BIC^c^** | **Adjusted BIC^d^** | **Entropy** | **P for VLMR^e^** | **P for aLMR^f^** | **Note** |
| **Summary score** | 1 | i s | free | free | 6922.806 | 6963.208 | 6931.475 | 0 |  |  |  |
|  | 1 | i s | free | same within class | 6928.47 | 6952.711 | 6933.671 | 0 |  |  |  |
|  | 2 | i s | free | free | 6877.354 | 6929.877 | 6888.624 | 0.458193 | 0.0016 | 0.0022 |  |
|  | 2 | i s | free | same within class | 6799.118 | 6839.521 | 6807.788 | 0.58479 | 0.005 | 0.0059 |  |
|  | 3 | i s | free | free | 6843.948 | 6908.592 | 6857.819 | 0.542055 | 0.0114 | 0.0138 |  |
|  | 3 | i s | free | same within class | 6760.89 | 6817.454 | 6773.028 | 0.655198 | 0.2116 | 0.22 |  |
|  | 4 | i s | free | free | 6842.694 | 6919.459 | 6859.166 | 0.612572 | 0.1323 | 0.1424 | 1 group 2% sample |
|  | 4 | i s | free | same within class | 6740.392 | 6813.117 | 6755.997 | 0.551534 | 0.0033 | 0.0039 |  |
|  | 5 | i s | free | same within class | 6726.819 | 6815.705 | 6745.892 | 0.611706 | 0.0039 | 0.0048 | 1 group 2.8% sample |
| Yellow marked model is the selected model.  ^a^ EORTC QLQ-C30: European Organisation for Research and Treatment of Cancer Quality of Life Questionnaire-Core 30.  ^b^ AIC: Akaike Information Criterion  ^c^ BIC: Bayesian Information Criterion  ^d^ Sample-size adjusted BIC  ^e^ VLMR: Vuong-Lo-Mendell-Rubin test  ^f^ aLMR: adjusted Lo-Mendell-Rubin test | | | | | | | | | | | |

| **eTable 3. Fit statistics for model comparison of EORTC-QLQ C30^a^ global quality of life and functional scales** | | | | | | | | | | | |
| --- | --- | --- | --- | --- | --- | --- | --- | --- | --- | --- | --- |
| **Health-related quality of life** | **Trajectory number** | **Latent variance** | **Latent mean** | **Residual variance** | **AIC^b^** | **BIC^c^** | **Adjusted BIC^d^** | **Entropy** | **P for VLMR^e^** | **P for aLMR^f^** | **Note** |
| **Global quality of life** | 1 | i s | free | free | 7664.293 | 7704.931 | 7673.197 | 0 |  |  |  |
|  | 1 | i s | free | same within class | 7660.68 | 7685.063 | 7666.022 | 0 |  |  |  |
|  | 2 | i s@0 | free | free | 7653.112 | 7697.814 | 7662.906 | 0.474092 | 0.1054 | 0.1195 |  |
|  | 2 | i s | free | same within class | 7623.803 | 7664.44 | 7632.706 | 0.51395 | 0.0002 | 0.0003 |  |
|  | 3 | i s@0 | free | free | 7652.838 | 7709.731 | 7665.303 | 0.525346 | 0.0905 | 0.0987 | 1.8% sample in one trajectory |
|  | 3 | i s@0 | free | same within class | 7623.744 | 7672.509 | 7634.428 | 0.385914 | 0.4408 | 0.4561 |  |
| **Physical function** | 1 | i s | free | free | 7525.2 | 7565.83 | 7534.1 | 0 |  |  |  |
|  | 1 | i s | free | same within class | 7521.36 | 7545.74 | 7526.7 | 0 |  |  |  |
|  | 2 | i s | free | free | 7441.93 | 7494.76 | 7453.51 | 0.82481 | 0.0009 | 0.0013 |  |
|  | 2 | i s | free | same within class | 7292 | 7332.63 | 7300.9 | 0.66159 | 0 | 0 |  |
|  | 3 | i s | free | free | 7415.87 | 7480.89 | 7430.11 | 0.72662 | 0.2859 | 0.3001 | 4.1% sample in one trajectory |
|  | 3 | i s | free | same within class |  |  |  |  |  |  | model not terminated normally |
|  | 2 | i s q@0 | free | free | 7430.955 | 7491.912 | 7444.311 | 0.830582 | 0.0084 | 0.0102 |  |
|  | 2 | i s q@0 | free | same within class | 7271.037 | 7319.802 | 7281.721 | 0.669697 | 0 | 0 |  |
| **Role function** | 1 | i s | free | free | 8306.102 | 8346.74 | 8315.006 | 0 |  |  |  |
|  | 1 | i s | free | same within class | 8307.16 | 8331.542 | 8312.502 | 0 |  |  |  |
|  | 2 | i s | free | free | 8229.352 | 8282.181 | 8240.927 | 0.787421 | 0.0017 | 0.0023 |  |
|  | 2 | i s | free | same within class |  |  |  |  |  |  | model not terminated normally |
|  | 3 | i s@0 | free | free | 8183.486 | 8240.379 | 8195.951 | 0.613731 | 0.0001 | 0.0001 |  |
|  | 4 | i s@0 | free | free |  |  |  |  |  |  | 5.5% sample in one trajectory |
| **Emotional function** | 1 | i s | free | free | 7625.833 | 7666.471 | 7634.737 | 0 |  |  |  |
|  | 2 | i s | free | same within class | 7498.772 | 7539.409 | 7507.675 | 0.572641 | 0 | 0 |  |
|  | 2 | i s@0 | free | free | 7570.938 | 7615.64 | 7580.732 | 0.751618 | 0.0001 | 0.0001 |  |
|  | 3 | i s@0 | free | free | 7555.357 | 7612.25 | 7567.822 | 0.67268 | 0.4069 | 0.4286 |  |
|  | 3 | i s | free | same within class |  |  |  |  |  |  | model not terminated normally |
| **Cognitive function** | 1 | i s | free | free | 7477.055 | 7517.693 | 7485.959 | 0 |  |  |  |
|  | 1 | i s | free | same within class | 7474.369 | 7498.752 | 7479.711 | 0 |  |  |  |
|  | 2 | i s | free | free |  |  |  |  |  |  | model not terminated normally |
|  | 2 | i s | free | same within class | 7240.581 | 7281.219 | 7249.485 | 0.691751 | 0 | 0 |  |
|  | 3 | i s | free | same within class | 7199.613 | 7256.506 | 7212.078 | 0.721983 | 0.0035 | 0.0043 | 7.9% sample in one trajectory |
| **Social function** | 1 | i s | free | free | 7972.364 | 8013.002 | 7981.268 | 0 |  |  |  |
|  | 1 | i s | free | same within class | 7974.186 | 7998.569 | 7979.528 | 0 |  |  |  |
|  | 2 | i s | free | free | 7906.561 | 7959.39 | 7918.136 | 0.769042 | 0.0005 | 0.0007 |  |
|  | 2 | i s | free | same within class |  |  |  |  |  |  | model not terminated normally |
|  | 3 | i s@0 | free | free | 7861.283 | 7918.176 | 7873.748 | 0.64059 | 0.007 | 0.0083 | 4.1% sample in one trajectory |
| Yellow marked model is the selected model.  ^a^ EORTC QLQ-C30: European Organisation for Research and Treatment of Cancer Quality of Life Questionnaire-Core 30.  ^b^ AIC: Akaike Information Criterion  ^c^ BIC: Bayesian Information Criterion  ^d^ Sample-size adjusted BIC  ^e^ VLMR: Vuong-Lo-Mendell-Rubin test  ^f^ aLMR: adjusted Lo-Mendell-Rubin test | | | | | | | | | | | |

| **eTable 4. Fit statistics for model comparison of EORTC-QLQ C30^a^** **symptom scales and items** | | | | | | | | | | | |  |
| --- | --- | --- | --- | --- | --- | --- | --- | --- | --- | --- | --- | --- |
| **Health-related quality of life** | **Trajectory number** | **Latent variance** | **Latent mean** | **Residual variance** | **AIC^b^** | **BIC^c^** | **Adjusted BIC^d^** | **Entropy** | **P for VLMR^e^** | **P for aLMR^f^** | **Note** | |
| **Fatigue** | 1 | i s | free | free | 7928.301 | 7968.938 | 7937.204 | 0 |  |  |  | |
|  | 1 | i s | free | same within class | 7928.514 | 7952.897 | 7933.856 | 0 |  |  |  | |
|  | 2 | i s | free | free | 7899.467 | 7952.296 | 7911.041 | 0.501758 | 0.0386 | 0.0448 |  | |
|  | 2 | i s | free | same within class | 7858.709 | 7899.346 | 7867.612 | 0.516869 | 0 | 0 |  | |
|  | 3 | i s | free | free | 7885.691 | 7950.712 | 7899.937 | 0.513765 | 0.107 | 0.1169 |  | |
|  | 3 | i s | free | same within class | 7844.538 | 7901.431 | 7857.003 | 0.611251 | 0.1936 | 0.2037 | 1 group 5.5% sample | |
|  | 4 | i s | free | free | 7884.05 | 7961.262 | 7900.967 | 0.510539 | 0.1765 | 0.1901 |  | |
|  | 4 | i s | free | same within class | 7841.25 | 7914.398 | 7857.277 | 0.433456 | 0.1143 | 0.1204 | 1 group 9.5% sample | |
| **Nausea** | 1 | i s | free | free | 7683.54 | 7724.18 | 7692.44 | 0 |  |  |  | |
|  | 1 | i s | free | same within class | 7688.45 | 7712.83 | 7693.79 | 0 |  |  |  | |
|  | 2 | i s | free | free | 7586.13 | 7638.96 | 7597.71 | 0.86913 | 0.0003 | 0.0005 |  | |
|  | 2 | i s | free | same within class | |  |  |  |  |  | model not terminated normally | |
|  | 3 | i s@0 | free | free | 7555.902 | 7612.795 | 7568.368 | 0.724402 | 0.1695 | 0.1823 | 1 group 3.2% sample | |
| **Pain** | 1 | i s | free | free | 7967.34 | 8007.98 | 7976.25 | 0 |  |  |  | |
|  | 1 | i s | free | same within class | 7985.83 | 8010.21 | 7991.17 | 0 |  |  |  | |
|  | 2 | i s | free | free | 7880.759 | 7933.588 | 7892.334 | 0.821039 | 0.0003 | 0.0004 |  | |
|  | 2 | i s | free | same within class | 7708.12 | 7748.758 | 7717.024 | 0.714211 | 0 | 0 |  | |
|  | 3 | i@0 s | free | free | 7859.507 | 7916.4 | 7871.972 | 0.810867 | 0.3793 | 0.396 | 1 group 6.5% sample | |
|  | 3 | i s | free | same within class | |  |  |  |  |  | model not terminated normally | |
| **Dyspnea** | 1 | i s | free | free | 8114.429 | 8155.021 | 8123.287 | 0 |  |  |  | |
|  | 1 | i s | free | same within class | 8112.726 | 8137.081 | 8118.041 | 0 |  |  |  | |
|  | 2 | i s@0 | free | free | 8059.673 | 8104.323 | 8069.416 | 0.752204 | 0.0036 | 0.0047 |  | |
|  | 2 | i s | free | same within class | 8017.059 | 8057.65 | 8025.916 | 0.577626 | 0.0003 | 0.0004 |  | |
|  | 3 | i s | free | free | 7991.576 | 8056.521 | 8005.747 | 0.958159 | 0.0887 | 0.098 |  | |
|  | 3 | i s | free | same within class | 8020.574 | 8077.402 | 8032.974 | 0.687007 | 0.0899 | 0.0964 | 1 group <1% sample | |
| **Insomnia** | 1 | i s | free | free | 8163.785 | 8204.422 | 8172.688 | 0 |  |  |  | |
|  | 1 | i s | free | same within class | 8160.13 | 8184.512 | 8165.472 | 0 |  |  |  | |
|  | 2 | i s@0 | free | free | 8094.864 | 8139.566 | 8104.658 | 0.798918 | 0.0308 | 0.0367 |  | |
|  | 2 | i s | free | same within class | |  |  |  |  |  | model not terminated normally | |
|  | 3 | i s | free | free | 7851.764 | 7916.785 | 7866.01 | 0.967151 | 0.0468 | 0.0528 |  | |
| **Appetite loss** | 1 | i s | free | free | 8278.289 | 8318.81 | 8287.076 | 0 |  |  |  | |
|  | 1 | i s | free | same within class | 8283 | 8307.313 | 8288.272 | 0 |  |  |  | |
|  | 2 | i s | free | free | 8160.219 | 8212.897 | 8171.643 | 0.798548 | 0.0003 | 0.0004 |  | |
|  | 2 | i s | free | same within class | |  |  |  |  |  | model not terminated normally | |
|  | 3 | i s | free | free | 8135.978 | 8200.811 | 8150.037 | 0.73193 | 0.2071 | 0.2222 | 1 group 4% sample | |
| **Constipation** | 1 | i s | free | free | 7724.408 | 7765.045 | 7733.311 | 0 |  |  |  | |
|  | 1 | i s | free | same within class | 7736.486 | 7760.868 | 7741.828 | 0 |  |  |  | |
|  | 2 | i s | free | free | 7528.353 | 7581.183 | 7539.928 | 0.988976 | 0.021 | 0.0249 | 1 group 8.1% sample | |
|  | 2 | i s | free | same within class | |  |  |  |  |  | model not terminated normally | |
| **Diarrhea** | 1 | i s | free | free | 8112.948 | 8153.516 | 8121.782 | 0 |  |  |  | |
|  | 1 | i s | free | same within class | 8126.021 | 8150.361 | 8131.321 | 0 |  |  |  | |
|  | 2 | i s@0 | free | free | 8071.374 | 8115.998 | 8081.091 | 0.732148 | 0.1912 | 0.2048 |  | |
|  | 2 | i s@0 | free | same within class | 8014.565 | 8047.019 | 8021.632 | 0.531737 | 0 | 0 |  | |
|  | 3 | i s@0 | free | free | 8058.009 | 8114.804 | 8070.377 | 0.646521 | 0.3865 | 0.3944 | 1 group 1.8% sample | |
|  | 3 | i s@0 | free | same within class | |  |  |  |  |  | model not terminated normally | |
| **Financial difficulty** | 1 | i s | free | free | 7754.66 | 7795.298 | 7763.564 | 0 |  |  |  | |
|  | 1 | i s | free | same within class | 7768.347 | 7792.73 | 7773.689 | 0 |  |  |  | |
|  | 2 | i@0 s | free | free | 7508.183 | 7552.885 | 7517.977 | 0.961697 | 0 | 0.0001 | 1 group 14.6% sample | |
|  | 2 | i s | free | same within class | |  |  |  |  |  | model not terminated normally | |
| Yellow marked model is the selected model.  ^a^ EORTC QLQ-C30: European Organisation for Research and Treatment of Cancer Quality of Life Questionnaire-Core 30  ^b^ AIC: Akaike Information Criterion  ^c^ BIC: Bayesian Information Criterion  ^d^ Sample-size adjusted BIC  ^e^ VLMR: Vuong-Lo-Mendell-Rubin test  ^f^ aLMR: adjusted Lo-Mendell-Rubin test | | | | | | | | | | | |  |
